# West African donkey’s liveweight estimation using body measurements

**DOI:** 10.14202/vetworld.2017.1221-1226

**Published:** 2017-10-12

**Authors:** Pierre Claver Nininahazwe, Adama Sow, Rakiswende Constant Roamba, Miguiri Kalandi, Hachi Dirir Ahmed, Georges Anicet Ouédraogo, Germain Jérôme Sawadogo

**Affiliations:** 1 Inter-States School of Veterinary Science and Medicine, Laboratory of Endocrinology and Radio-Immunology, BP. 5077, Dakar Fann, Senegal; 2 Directorate General of Veterinary Services, 03 BP. 7026, Ouagadougou 03, Burkina Faso; 3 Department of Livestock and Veterinary Services Boulaos, BP. 297, Djibouti City, Republic of Djibouti; 4 Laboratory of Teaching and Research in Animal Health and Biotechnology, Polytechnic University 01 BP. 1091 Bobo-Dioulasso 01, Burkina Faso

**Keywords:** body measurements, donkey, estimated liveweight, West Africa

## Abstract

**Aim:**

The objective of this study was to determine a formula for estimating the liveweight in West African donkeys.

**Materials and Methods:**

Liveweight and a total of 6 body measurements were carried out on 1352 donkeys from Burkina Faso, Mali, Niger, and Senegal. The correlations between liveweight and body measurements were determined, and the most correlated body measurements with liveweight were used to establish regression lines.

**Results:**

The average weight of a West African donkey was 126.0±17.1 kg, with an average height at the withers of 99.5±3.67 cm; its body length was 104.4±6.53 cm, and a heart girth (HG) of 104.4±6.53 cm. After analyzing the various regression lines and correlations, it was found that the HG could better estimate the liveweight of West African donkeys by simple linear regression method. Indeed, the liveweight (LW) showed a better correlation with the HG (R^2^=0.81). The following formulas (Equations 1 and 2) could be used to estimate the LW of West Africa donkeys.

Equation 1: Estimated LW (kg) = 2.55 × HG (cm) - 153.49;

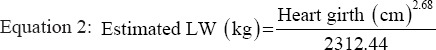

**Conclusion:**

The above formulas could be used to manufacture weighing tape to be utilized by veterinary clinicians and farmers to estimate donkey’s weight in the view of medication and adjustment of load.

## Introduction

In West Africa, donkey contributes significantly to socioeconomic life. That animal is used in both rural and urban areas. Due to the poor mechanization of agriculture and small farms, traction animals such as oxen, horses, and donkeys are needed for crop production. A donkey is an easy-care and docile animal, and its use as animal traction allows the farmer to increase their crop production surface. In arid areas, a donkey, equipped with an appropriate equipment, can pump 3600 l of water into a 10-m deep well in 20 min [1].

In urban areas, garbage is collected by carts with donkey traction. In addition, the donkey is used for the transport of various goods, in particular, building materials. In Burkina Faso, Tapsoba [2] reported that this activity generates an average of 5-10 US$/day to the donkey owners.

Despite that socioeconomic importance, there is no measuring tape for estimating the liveweight (LW) of West Africa donkey. Although formulas for estimating donkey weights are established in Zimbabwe, Kenya, Morocco, and Nigeria [3-6], these formulas could not be fully applicable to the West African donkey, due to zootechnical, genetic, and environmental specificities.

While estimating LW for animals such as donkeys is essential for good health care and estimating traction power (instantaneous power) [7], weighing with a balance is the most accurate way. However, in rural areas, weighing machines are not often available because of the cost of this equipment.

Therefore, body measurements are an alternative technique that is easy to implement because estimating LW using body measurements is practical, quicker, easier, and cheaper in rural areas where breeders have limited resources [8]. To contribute to the welfare of donkeys in West Africa, the objective of this study was to establish a LW prediction equation using body measurements.

## Materials and Methods

### Ethical approval

This study was approved by Animal Ethical Committee of the Inter-states Veterinary School of Dakar. All donkeys were sampled with informed consent of their owners and with respect for animal welfare.

### Animals’ sampling

The sample used in this study consisted of 1352 donkeys including 1001 males and 351 non-pregnant females from Burkina Faso, Mali, Niger, and Senegal. LW and body measurements were carried out on each donkey (Table-1).

**Table-1:** Characterization of the donkey population of the study.

Country	Number	Males	Females	Mean age±SD
Burkina Faso	489	367	122	6.58±3.7
Mali	292	200	92	7.06±3.9
Niger	281	247	34	7.58±3.2
Senegal	290	187	103	4.74±2.6
Total	1352	1001	351	6.49±3.6

SD=Standard deviation

### Body measurements

**Figure-1:**
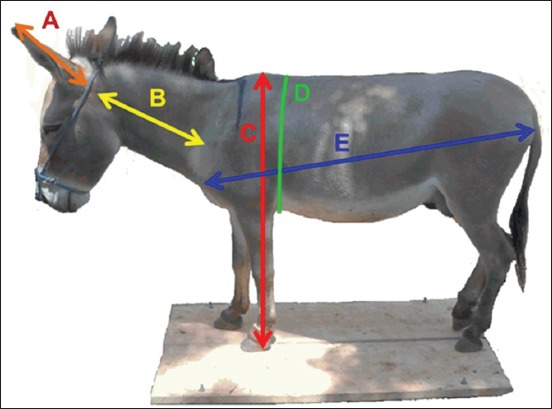
Body measurements used in this study including height at the withers, body length (BL), heart girth (HG), neck length (NL), and ears length (EL). (a) EL, (b) NL, (c) height at withers, (d) HG, (e) BL.

For each animal, the LW was determined after its immobilization on the weighing scale (Ruddweigh KM-2E Electronic Weighing System®). Body measurements included height at the withers (HW), body length (BL), heart girth (HG), neck length (NL), and ears length (EL) (Figure-1). All measurements were made when the animal is calm, standing squarely on a flat and horizontal surface with a normal head port.

The HW determined with the aid of the measuring rod corresponds to the distance between the apex of the withers and the floor. The BL measured with a metric tape is the distance between the tip of the shoulder (olecranon process) and the tip of the buttock (ischium tuberosity). The HG corresponds to the measurement passing vertically behind the tourniquet and at the level of the passage of the straps. It was determined using the metric tape when the donkey is expiring. The NL is measured over the distance from the base of the scapula to the base of the mandible. The head must be straight when measuring this parameter. The length of the left and right ear (LRE and LLE) was measured using the metric tape. This length extends from the implantation base of the ear with the head to the tip.

### Statistical analysis

LW was considered as a variable to be explained (dependent variable), the explanatory variables being HW, BL, HG, NL, LRE, and LLE. The statistical analyses were carried out using the Statistical Package for the Social Sciences (SPSS) 23.0 software. The averages and standard deviations of all variables were calculated. In addition, the univariate general linear model was used to determine the effects of variables (country, sex, or age) on LW and body measurements. Depending on sex or age, correlation coefficients (r) between LW and body measurements were calculated. The determination coefficients (R^2^) of the LW prediction equations were determined using simple and multiple linear regressions with gender or age. The different LW prediction formulas were compared.

## Results

### Body measurements

Table-2 summarizes the different body measurements of donkeys by country of origin. The mean LW was 118.1±21.7 kg. The country of origin of the animals had a significant influence on the LW of these animals (p<0.05). As the body weight, most body measurements varied significantly according to the geographical origin of the donkeys. The average HW, which is 98.37±4.9 cm for all donkeys in the 4 countries of West Africa, varied significantly (p<0.05) from 100.38±3.9 cm for donkeys from Niger to 97.46±5.5 cm for those of Burkina Faso.

**Table-2:** Values of body parameters by country of origin.

Variable	Burkina Faso (n=489)	Mali (n=292)	Niger (n=281)	Senegal (n=290)	Total
LW	113.9±23.0^a^	116.4±23.9^a^	121.2±13.5^b^	123.7±22.4^b^	118.1±21.7
HG	105.0±8.28^a^	105.1±8.32^a^	107.9±5.25^b^	108.9±7.01^b^	106.5±7.6
BL	103.9±7.78^a^	104.8±7.81^a^	100.3±5.89^b^	100.0±10.60^b^	102.6±8.4
Height	97.5±5.48^a^	98.1±5.13^a^	100.4±3.85^b^	98.1±4.14^c^	98.4±4.9
NL	28.7±2.58^a^	28.4±2.19^a^	35.1±4.41^b^	30.9±4.88^c^	30.4±4.3
LRE	24.6±1.42^a^	24.1±1.21^b^	28.2±1.87^c^	26.5±1.61^d^	25.6±2.2
LLE	25.6±1.39^a^	24.2±1.21^b^	28.6±1.74^c^	26.5±1.73^d^	25.7±2.2

The averages of the in-line variables with different letters (a, b, c, and d) are significantly different (p<0.05). LW=Liveweight, BL=Body length, HG=Heart girth, NL=Neck length, LLE=Left ear length, LRE=Length right ear

### Variation factors of body measurements

LW and body measurements varied significantly (p<0.05), depending on the age and sex classes of the donkeys (Tables-3 and 4). Thus, donkeys <3 years of age had an average LW (87.14 kg) less than that of older donkeys (122.67 kg). Similarly, males were heavy as females. However, the ears had on average the same lengths in males as females.

**Table-3:** Measurement values by age group.

Variable	<3 years (n=175)	≥3 years (n=1177)	p value
LW	87.1±26.9	122.7±16.5	<0.001
HG	95.7±10.99	108.0±5.48	<0.001
BL	92.4±11.04	104.0±6.74	<0.001
Height	92.1±7.38	99.2±3.66	<0.001
NL	27.1±3.49	31.0±4.26	<0.001
LRE	24.5±2.06	25.8±2.15	<0.001
LLE	24.5±1.93	25.9±2.24	<0.001

LW=Liveweight, BL=Body length, HG=Heart girth, NL=Neck length, LLE=Left ear length, LRE=Length right ear

**Table-4:** Measurement values by sex.

Variables	Female (n=351)	Male (n=1001)	p value
LW	115.4±26.1	119.0±19.9	0.007
HG	105.3±9.45	106.9±6.89	0.001
BL	100.9±10.0	103.1±7.66	<0.001
Height	96.9±5.57	98.9±4.61	<0.001
NL	29.3±3.55	30.9±4.55	<0.001
LRE	25.5±2.13	25.7±2.21	0.151
LLE	25.5±2.21	25.8±2.26	0.982

LW=Liveweight, BL=Body length, HG=Heart girth, NL=Neck length, LLE=Left ear length, LRE=Length right ear

### Correlations between LW and body measurements

The correlations between LW and body measurements were determined by sex or age of the animals. All correlations were statistically significant (p<0.05). This relationship was more marked with the HG (r=0.90), moderately marked with the HW (r=0.72) and the BL (r=0.62), and weakly marked with the NL (r=0.37), LRE (r=0.31), and LLE (r=0.29) (Table-5).

**Table-5:** Correlations between LW and body measurements.

Classes	HG	BL	HG	NL	LRE	LLE
Male	0.90	0.61	0.7	0.33	0.29	0.27
Female	0.89	0.64	0.76	0.51	0.35	0.33
<3 years	0.93	0.74	0.82	0.62	0.57	0.54
≥3 years	0.82	0.41	0.53	0.2	0.16	0.16
Total	0.90	0.62	0.72	0.37	0.31	0.29

LW=Liveweight, HW=Height at the withers, BL=Body length, HG=Heart girth, NL=Neck length, LLE=Left ear length, LRE=Right ear length, r=Correlation coefficient

### Prediction equations of LW

The assessment of the regression possibilities was carried out by the representation of the point clouds between the LW and the measures most correlated with the LW, namely, HG, the HW, and the BL.

**Figure-2:**
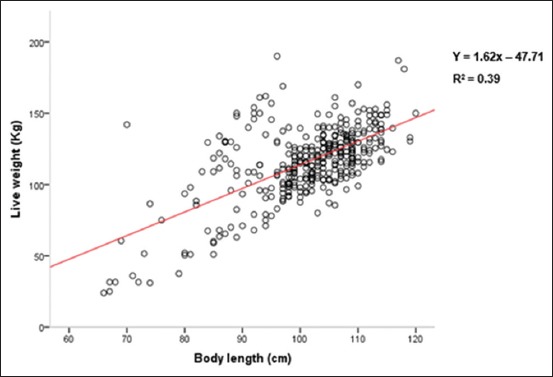
Point cloud between liveweight (LW) and body length (BL). The expression of the LW as a function of the BL showed a strong dispersion of the cloud of points.

**Figure-3:**
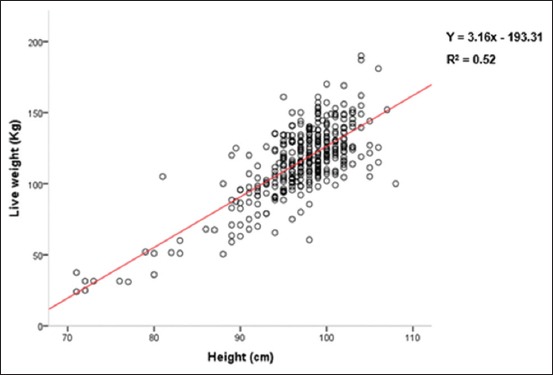
Point cloud between the liveweight (LW) and the height at withers. The expression of the LW according to the height at the withers showed an intermediate situation of the cloud of points between the dispersion and the linearity.

**Figure-4:**
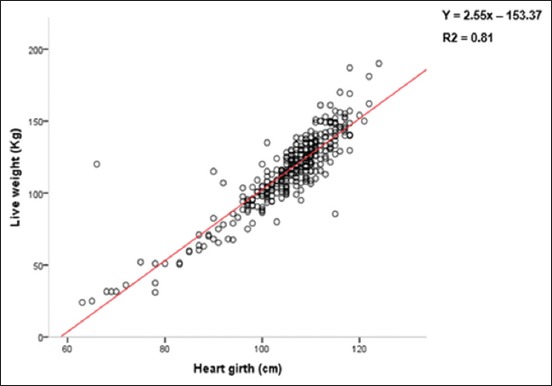
Point cloud between liveweight (LW) and heart girth. The expression of LW as a function of the thoracic perimeter showed an almost linear distribution of the cloud of points.

The expression of the LW according to these measurements is illustrated by different graphs (Figures-2-4). These figures show a strong dispersion of the point cloud between LW and BL (Figure-2), an intermediate situation between LW and HW (Figure-3), and an almost linear distribution of the point cloud between LW and HG (Figure-4).

When the predictive LW model is based on BL, the coefficient of determination (R^2^) is 0.39. Furthermore, the coefficient of determination (R^2^) increases from 0.52 to 0.81 when the predictive model of the LW is determined from the HW and the HG, respectively. Consequently, among these explanatory variables (HG, BL, and HW), only the HG remains a better estimator of the LW in all the sampled animals. Precision is improved in multiple regressions (estimates according to several explanatory variables) compared to simple regressions. Thus, the association of BL with HG slightly increases the accuracy of the LW estimation (Tables-6 and 7).

**Table-6:** Equations of simple and multiple linear regressions.

Classes	n	Equations with 1 variable		Equations with 2 variables	
	
		p=f (HG)	R^2^	p=f (HG. BL)	R^2^
Male	1001	2.61HG-159.78	0.81	2.39HG+0.33BL−170.97	0.82
Female	351	2.47HG-144.44	0.79	2.28HG+0.28BL−152.43	0.8
<3 years	175	2.29HG-131.87	0.87	2.10HG+0.26BL−137.44	0.88
≥3 years	1177	2.49HG-146.20	0.68	2.36HG+0.30BL−162.58	0.69
Total	1352	2.55HG-153.49	0.81	2.34HG+0.31BL−163.13	0.82

p=Weight calculated from the equations, R^2^=Coefficient of determination. HG=Heart girth, BL=Body length

**Table-7:** Log equations of simple and multiple linear regressions.

Classes	n	Log equations with 1 variable		Log equations with 2 variables	
	
		p=f (HG)	R^2^	p=f (HG, BL)	R^2^
Male	1001	HG^2.672^/e^7.718^	0.83	(HG^2.434^*BL^0.345^)/e^8.202^	0.85
Female	351	HG^2.682^/e^7.767^	0.83	(HG^2.365^*BL^0.445^)/e^8.339^	0.85
<3 years	175	HG^2.917^/e^8.879^	0.89	(HG^2.641^*BL^0.359^)/e^9.244^	0.89
≥3 years	1177	HG^2.155^/e^5.286^	0.66	(HG^2.038^*BL^0.256^)/e^5.928^	0.68
Total	1352	HG^2.69^/2322	0.81	2.34HG+0.31BL−163.13	0.82

p=Weight calculated from the equations, R^2^=Coefficient of determination. HG=Heart girth, BL: Body length

Therefore, the following LW prediction equations were issued:

Equation 1: Estimated LW (kg) = 2.55 × HG (cm) - 153.49;


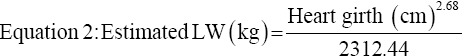


## Discussion

In this study, there were more males (74%) than females, and the majority of sampled donkeys (87.06%) were older than or equal to 3 years. This would be due to the fact that a donkey can start working from the time that they are 2.5-3 years in a very gradual and moderate way so as to adapt the effort (frequency, duration, and intensity) to its age [9]. In addition, the breeding of the donkeys starts with animals aged at least 3 years [10], and farmers buy donkeys which are ready to support traction, i.e., at least 3 years old.

This study revealed that the average LW of donkeys in West Africa is 118.08 kg. Hence, donkeys in West Africa weigh less than donkeys elsewhere in Central Africa, Southern Africa, and North Africa. The donkeys of Cameroon, Zimbabwe, and Morocco average LW were 123.2 kg, 142 kg, and 135 kg, respectively [3,4,11]. The country of origin of the study animals had a significant effect on the LW of these animals and the values of the body measurements done on these same animals. This could be due to several factors such as environment, genetics, and diet.

There was a significant difference between the values of all the variables (LW and measurements) according to the age classes of the animals. This would be due to the fact that the physiological evolution from one age group to another leads to an increase in weight and morphological growth. These findings corroborate those of Roamba [12] and Kaboré [13], whereby young animals, adults, and elderly animals do not exhibit the same body parameters, and these increases concomitantly with the age of the animals. The effect of sex was significant on the LW of the study animals and the values of all measured measurements except the EL. These results confirm those of Kaboré [13], and this situation could be explained by a sexual dimorphism.

The correlations between LW and different measurements were all significant (p<0.001). Regardless of the sex or age of the donkey, the HG was the only measure highly correlated with LW. For all animals, the correlation coefficient is 0.90 with the HG. These results confirm the work carried out in Zimbabwe by Nengomasha *et al*. [3], those undertaken in Kenya by Pearson and Ouassat [5] and those carried out in Nigeria by Hassan *et al*. [6]. Indeed, these authors reported that the HG is the most correlated mensuration to estimate the LW in donkeys. Furthermore, Aluja *et al*. [14] reported that the best models for predicting the LW of donkeys in central Mexico would be those using the HG.

In simple regression between LW and body measurements, the coefficients of determination found for all the animals studied are 0.81 with the HG and 0.39 with the BL. This demonstrates, once again, the strong correlation and high accuracy with which the LW of the donkeys can be estimated using the HG. These results are different from those of Hassan *et al*. [6] and Nengomasha *et al*. [3]. In fact, Hassan *et al*. [6] reported coefficients of determination (R^2^) of 0.99 and 0.72, respectively, with the HG and BL on donkeys in north-western Nigeria. As for Nengomasha *et al*. [3], they reported a coefficient of determination (R^2^) of 0.86 with the HG in donkeys in western Zimbabwe. This difference is due to the effects of heterogeneity, the environment, and the sampling of animals.

As the number of variables taken into account increases, the predictive accuracy of the LW increases. The association of BL with HG slightly increased the coefficient of determination (R^2^) from 0.81 to 0.82 in the LW estimate in this study. These results are in agreement with the work of Pearson and Ouassat [4] who reported a better prediction equation for the LW of draft donkeys in Morocco according to two variables, the HG and the BL.

Moreover, in mathematics, Falissard [15] reported that the total correlation coefficient of the multiple regression of y on x and z is always greater than or equal to that of the simple regression on one of the two variables, confirming the results of Landais [16] that the information provided by two variables is richer than that provided by a single variable. As a consequence, the error in the weight estimation by the simple linear equations would be greater than that of the multiple linear regressions proposed in this study. Weights estimated from multiple linear equations are closer to the observed weights than those calculated from a simple linear equation. However, equations formulated solely from the HG remain simple, reliable, and easy to use in predicting the LW of the animal. Subsequently, the equation: LW (kg) = 2.55 × (HG [cm]) - 153.49 was chosen to estimate the weight of West African donkeys.

## Conclusion

From the results of the present study, it was concluded that the Equations 1 and 2 could be used to estimate the LW of West Africa donkeys. Thereby, a measuring tape for estimating the LW of West African donkey could be produced and made available for veterinary clinicians and farmers to ensure a better health care and the adjustment of traction power.

## Authors’ Contributions

PCN, AS, RCR, and HDA contributed to study design and field data collection. PCN, AS, MK, GAO, and GJS provided guidance for data analysis. All authors participated to the redaction of the manuscript. All authors have read and approved the final manuscript.
